# Supplementary material for: De novo assembly and characterization of the transcriptome of Morchella esculenta growth with selenium supplementation
Source: PeerJ. 2024 May 31;12:e17426. doi: 10.7717/peerj.17426 (PMC11146319; doi:10.7717/peerj.17426)
Supplement: Supplemental Information 1 [file peerj-12-17426-s001.zip › Attachment materials/Supplementary tables.docx]

Table S1 The number of sequenced and mapped reads of genome-wide transcription analysis for each sample.

| Sample | Total Clean Reads | Total Mapping (%) | GC content  (%) | Clean Reads  Q20 (%) | Clean Reads  Q30 (%) |
| --- | --- | --- | --- | --- | --- |
| CK -1 | 22880250 | 91.08 | 50.97 | 96.08 | 91.18 |
| CK -2 | 46289028 | 86.02 | 48.93 | 96.85 | 92.06 |
| CK -3 | 44263813 | 85.55 | 48.91 | 96.71 | 91.94 |
| MSe5-1 | 22136133 | 89.42 | 49.13 | 95.75 | 90.53 |
| MSe5-2 | 22588714 | 90.27 | 49.98 | 96.43 | 91.56 |
| MSe5-3 | 24907802 | 90.56 | 49.81 | 96.01 | 90.9 |
| MSe10-1 | 22486541 | 90.13 | 48.86 | 96.16 | 91.11 |
| MSe10-2 | 22011872 | 90.36 | 48.8 | 96.36 | 91.47 |
| MSe10-3 | 20455678 | 90.59 | 49.26 | 96.18 | 91.26 |

Table S2 Differentially expressed genes in *M. esculenta* under selenite treatment by RNA-seq and q-PCR analysis

| Gene ID | Description | Primer | Relative abundance log_2_ (FC) in MSe5 | | Relative abundance log_2_ (FC) in MSe10 | |
| --- | --- | --- | --- | --- | --- | --- |
|  |  |  | RNA-Seq | qPCR | RNA-Seq | qPCR |
| NODE_1429 | MFS general substrate transporter | F: 5'-GCGTCTCCTCCCGTTCATTT-3'  R:5'-CGGCACTTCATTGGGTTCTA-3' | 5.15 | 4.65 | 5.83 | 5.12 |
| NODE_17890 | RTA1 like protein | F: 5'-TACATCTTCCGCCTCATCTCCA-3'  R:5'-TCGTCCAGTGATACCTGCCATAC-3' | 5.15 | 4.32 | 5.86 | 4.87 |
| NODE_13398 | TLD-domain-containing protein | F: 5'-CTGGTCCCGTGTCTGTTGGT-3'  R:5'-CGCGAGGATGATACTGTCTGG-3' | 5.26 | 5.11 | 6.11 | 5.48 |
| NODE_12777 | adenosine/AMP deaminase | F: 5'-GGGCGATGAGGCTAACAATG-3'  R:5'-CAGTAGGTAACAAGTACCACGAAT-3' | 5.34 | 4.76 | 6.55 | 5.87 |
| NODE_1467 | ARM repeat-containing protein | F: 5'-GACCGAACGCACCAATACAG-3'  R:5'-TCCGTGGAGCAGCAGTTGTA-3' | -5.43 | -4.72 | -5.47 | -4.85 |
| NODE_576 | hypothetical protein | F: 5'-GCCCTATTCGTTATGGTAGTGTTG-3'  R:5'-TGCTGCTGTTGAGTTTGCTGTT-3' | -5.55 | -4.88 | -5.77 | -5.21 |
| NODE_3248 | Importin subunit alpha | F: 5'-GCCCTCCTTTCCCTTCTTGG-3'  R:5'-ACCTCCGCTGGTTGCGTTAG-3' | -5.68 | -5.23 | -6.07 | -5.47 |
